# Thiopurine methyltransferase genotype and activity cannot predict outcomes of azathioprine maintenance therapy for antineutrophil cytoplasmic antibody associated vasculitis: A retrospective cohort study

**DOI:** 10.1371/journal.pone.0195524

**Published:** 2018-04-09

**Authors:** Arno C. Hessels, Abraham Rutgers, Jan Stephan F. Sanders, Coen A. Stegeman

**Affiliations:** 1 Department of Internal Medicine, Division of Nephrology, University of Groningen, University Medical Center Groningen, Groningen, The Netherlands; 2 Department of Rheumatology and Clinical Immunology, University of Groningen, University Medical Center Groningen, Groningen, The Netherlands; Radboud university medical center, NETHERLANDS

## Abstract

**Objective:**

Azathioprine is a widely used immunosuppressive drug. Genetic polymorphisms and activity of the enzyme thiopurine methyltransferase (TPMT) have been associated with azathioprine efficacy and toxicity in several populations. We investigated whether these associations also exist for ANCA associated vasculitis (AAV) patients, who receive azathioprine maintenance therapy after remission induction with cyclophosphamide.

**Methods:**

207 AAV patients treated with cyclophosphamide induction and azathioprine maintenance therapy were included and followed for 60 months. *TPMT* genotype and tertiles of TPMT activity were compared to relapse free survival and occurrence of adverse events, particularly leukopenia. Multivariable regression was performed to account for confounders.

**Results:**

In univariable analysis, relapse free survival was not significantly associated with *TPMT* genotype (P = 0.41) or TPMT activity (P = 0.07), although it tended to be longer in lower tertiles of TPMT activity. There was no significant association of TPMT genotype and activity with occurrence of any adverse event. In multiple regression, leukocyte counts at the end of cyclophosphamide induction were related to risk of leukopenia during azathioprine therapy [P<0.001; OR 0.54 (95% CI 0.43–0.68)] and risk of relapse during follow-up [P = 0.001; HR 1.17 (95% CI 1.07–1.29)] irrespective of TMPT genotype or activity.

**Conclusion:**

TPMT genotype and activity were not independent predictors of relapse, and could not predict leukopenia or other adverse effects from azathioprine. Leukocyte counts after cyclophosphamide induction were related to both outcomes, implying a greater influence of cyclophosphamide response compared to azathioprine and TPMT in AAV patients.

## Introduction

Anti-neutrophil cytoplasmic antibody (ANCA)-associated vasculitis (AAV) refers to a group of primary small-vessel vasculitides. The most common forms are granulomatosis with polyangiitis (GPA, formerly Wegener’s Granulomatosis) and microscopic polyangiitis (MPA).[1] Induction treatment with cyclophosphamide, rituximab or mycophenolate mofetil combined with corticosteroids can achieve remission in most AAV patients and reduce mortality, but is associated with considerable toxicity.[2–4] For this reason, patients switch to less toxic maintenance therapy after achieving remission, most frequently azathioprine.[3–5] Even with azathioprine maintenance therapy, there is a risk of potentially severe adverse effects, most frequently leukopenia and infection.[5,6] In recent years, there has been increasing interest in personalised medicine whereby treatment is adjusted based upon characteristics of an individual patient, thereby optimizing efficacy and reducing toxicity.

Azathioprine and its metabolite 6-mercaptopurine are converted via several enzymatic steps into 6-thioguanine nucleotides (6-TGN), the active metabolites responsible for the immunosuppresive effect and myelotoxicity. The enzyme thiopurine methyltransferase (TPMT) methylates several metabolites along the enzymatic pathway, thereby reducing the amount of 6-TGN formed.[7,8] Several polymorphisms of the gene encoding TPMT have been identified, each resulting in decreased activity of the enzyme.[7–9] Approximately 89% of Caucasians are homozygous for wildtype *TPMT* alleles corresponding with normal or high activity.11% carry a wildtype and a variant allele corresponding with intermediate TPMT activity. Very few individuals (0.3%) are homozygous or compound heterozygous for variant alleles resulting in absence of TPMT activity.[8,10]

Studies in several populations, mainly inflammatory bowel disease (IBD), have shown that *TPMT* variant alleles and lower TPMT activity are associated with a higher risk of bone marrow toxicity.[7,11–13] Patients carrying two variant *TPMT* alleles are especially at risk for severe myelotoxicity[11] and require either a 10-fold lower dose or alternative therapy (e.g. methotrexate).[6,8,14] For patients with intermediate TPMT activity carrying one variant allele, more controversy exists. Several meta-analyses have shown an increased risk of myelotoxicity in these patients.[12,15] While clinical trials did not find a significant reduction of toxicity when adjusting azathioprine or 6-mercaptopurine dose on TPMT genotype or activity,[14,16,17] post-hoc analysis showed a significant reduction of myelotoxicity within carriers of a variant allele.[14]

The aforementioned studies mainly involve patients with IBD, who receive azathioprine as their main treatment drug. Since AAV patients receive azathioprine after an induction phase with cyclophosphamide,[4] the influence of TPMT might be smaller and less relevant in this population.

The aim of this study was to see whether TPMT genotype and activity are associated with bone marrow toxicity and risk of relapse in AAV patients treated with azathioprine maintenance therapy. We expanded on an earlier study in our population[18] by taking into account the influence of cyclophosphamide induction therapy on these outcomes.

## Patients and methods

### Patients

For this retrospective cohort study, 377 patients, diagnosed with GPA, MPA or Renal Limited Vasculitis (RLV) between September 1984 and August 2013 in the University Medical Center Groningen (UMCG) and treated with oral cyclophosphamide following diagnosis, were considered for inclusion. Patients were included if they switched to azathioprine after induction of remission and had a follow-up of at least a year. All patients have given written informed consent according to the Declaration of Helsinki for participation in a large cohort study investigating biomarkers (including TPMT) in relation to disease outcome in AAV. Ethical approval for the study was granted by the local Medical Ethical Committee of the University Medical Center Groningen (NL29354.042.10).

### Treatment protocol

Following diagnosis, all patients were treated with oral cyclophosphamide (1.5–2.0 mg/kg/day) combined with prednisolone (1mg/kg/day, max 60mg/day). Prednisolone dose was reduced according to a standard schedule (S1 Table). After 3 months of stable remission, all patients switched to maintenance therapy with azathioprine. The starting dose was a conversion from cyclophosphamide dose to the same azathioprine dose. The target azathioprine dose was 1.5–2.0mg/kg/day. Starting 12 months after diagnosis, azathioprine dose was reduced by 25 mg/day every 3 months. Leukocyte counts were measured 1 week after starting azathioprine and at least every 4 weeks thereafter. During treatment,cyclophosphamide and azathioprine dose were adjusted based on leukocyte counts (goal: leukocytes ≥4.0*10^9^/l) in accordance with the CYCAZAREM protocol,[5] and occurrence of infections.

### Data collection

All information was collected from the patients’ records. For all patients, demographic, disease and treatment characteristics, as well as clinical outcome data were registered. Diagnosis was based on the 2012 Chapel Hill Consensus Conference definitions.[1] Disease activity at diagnosis was scored using the Birmingham Vasculitis Activity Score 1 (BVAS-1).[19] Patients were screened for the presence of ANCA using indirect immune fluorescence (IIF), and ANCA-specificity was determined using ELISA.

The primary endpoints of the study were relapse-free survival in months and leukopenia. Relapse was defined as new or worsening disease activity requiring dose increase or switch of immunosuppressive medication. Leukopenia was defined as leukocyte count <4.0*10^9/l.[20] Secondary categorical endpoints were moderate leukopenia (leukocyte count <3.0*10^9/l),[20] macrocytic anemia (Hb <7.5 for females and <8.0 for males; MCV>96fl), hepatotoxicity (ASAT and/or ALAT >2x upper limit of normal, or AF >125 U/l), infection (requiring hospitalisation and/or antibiotics, or opportunistic *e*.*g*. CMV, VZV, HSV, and/or pneumocystis jirovecii pneumonia). These endpoints were scored if they occurred at any time during azathioprine therapy. Secondary continuous endpoints were leukocyte counts 3,6,9 and 12 months after switch to azathioprine, and the [leukocyte(*10^9^/l)]*[azathioprine(mg/kg/d)] product 3,6,9 and 12 months after switch as a measure of sensitivity for azathioprine-induced bone marrow depression.[18] Diagnosis, ANCA specificity, age at diagnosis, baseline serum creatinine, co-trimoxazole use at switch to azathioprine (none, prophylactic or therapeutic dose), leukocyte count at switch and duration of azathioprine therapy were registered for their potential influence on relapse. Factors registered for their potential influence on risk of leukopenia include prednisolone dose at switch, cyclophosphamide dose at switch, leukocyte count at switch and azathioprine dose at switch. Prednisolone dose during azathioprine therapy was registered to account for its influence on leukocyte counts.

### Measurement of *TPMT* genotype and TPMT activity

Four variants of the *TPMT* gene, located on chromosome 6, were determined using PCR, as described by Yates et al.[9] The genetic variants were TPMT*2 (G→C translocation at nucleotide 238), TPMT*3A (460G→A and 719A→G), TPMT*3B (460G→A), and TPMT*3C (719A→G).

TPMT activity was determined by adding 6-thioguanine to human erythrocytes in vitro, and measuring the amount of 6-methylthioguanine formed (TPMT catalyses this reaction), expressed in nmol 6-methylthioguanine formed per gram haemoglobin per hour (nmol/gHb/hr).[21] In the majority of patients (67%), TPMT genotype and activity were measured after starting azathioprine treatment. In some patients (33%), these were measured before starting azathioprine. The date of blood withdrawal for TPMT measurement was registered for all patients.

### Statistics

Statistical analysis was done using SPSS Statistics 22 (IBM Corporation, New York, US).

Data are shown as median + interquartile range (IQR) or number + percentage. A two-sided P<0.05 was considered statistically significant. Univariate analysis was performed for *TPMT* genotypes and tertiles of TPMT activity (tertiles determined based on equal numbers of patients per group) using a Log Rank test for relapse free survival (up to 60 months after diagnosis), Fisher’s exact test or Chi Square test for risk of adverse events, and Mann-Whitney or Kruskal-Wallis test for leukocyte count and [leukocyte]*[azathioprine] product. Multivariate analysis was performed with relapse-free survival, risk of leukopenia and leukocyte counts as outcome variable, using Cox regression, logistic regression and linear regression, respectively. Possible predictors in the analysis were *TPMT* genotypes, tertiles of TPMT activity and potential influencing factors for the respective outcomes mentioned under ‘data collection’. A forward stepwise model was used, where variables were included as covariates based on a univariate P<0.05 and excluded on a multivariate P-value >0.10. Non-proportional hazards for predictors in Cox regression were accounted for by adding a time-by-predictor interaction variable to the model.[22]

## Results

### Patients and TPMT genotype and activity

**Fig 1:**
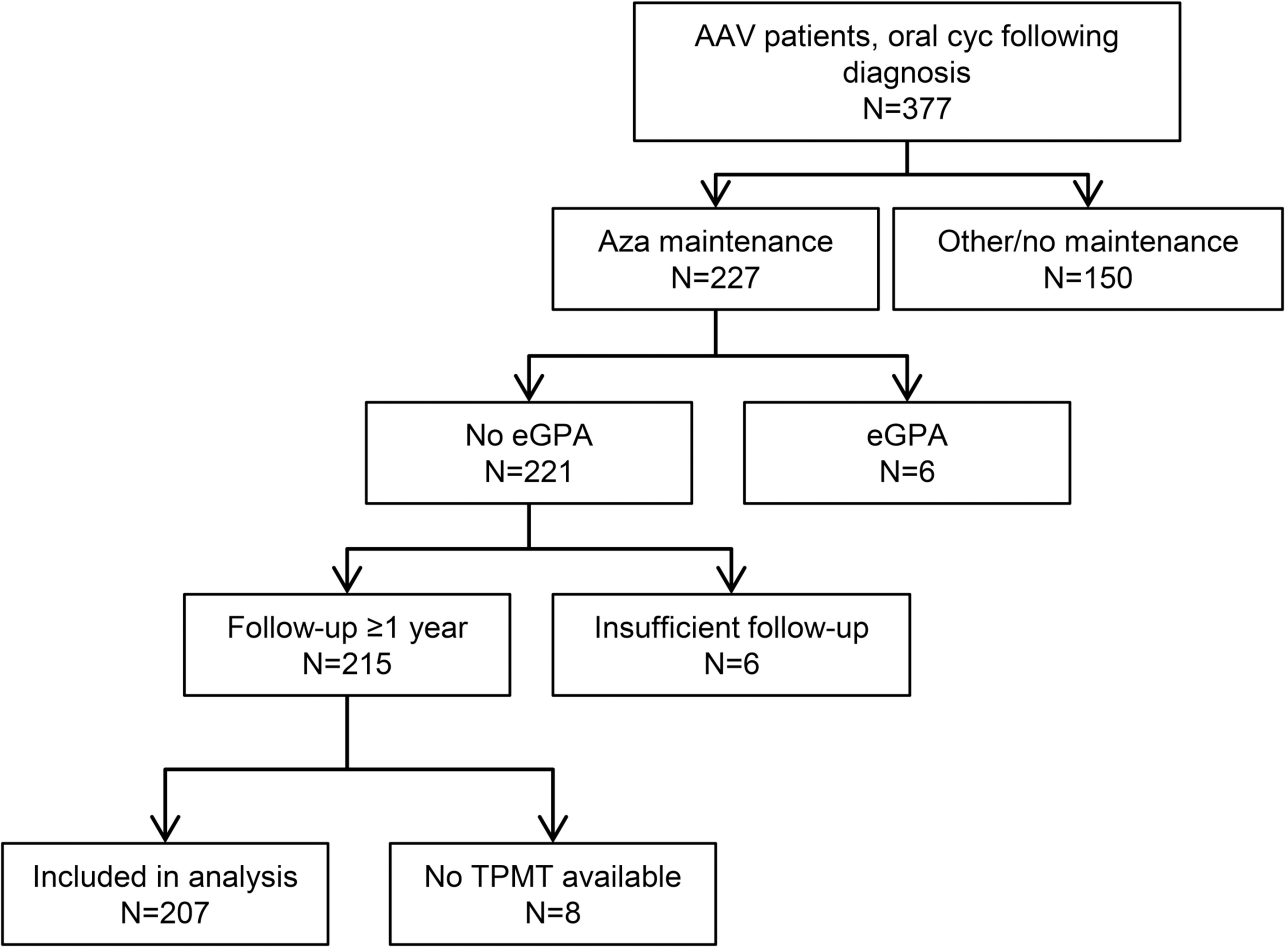
Flow-chart of patient selection.

207 patients were included in the analysis.(Fig 1) Demographic and disease characteristics, as well as distribution of *TPMT* genotypes and TPMT activity, are shown in Table 1.

**Table 1:** Patient characteristics.

Characteristics	N (%)/mean (SD)/median (IQR)
Female	94 (45%)
Age at diagnosis (years)	57 (46–66)
Diagnosis GPA	152 (73%)
PR3-ANCA	150 (73%)
BVAS at diagnosis	18 (13–24)
Serum creatinine at baseline (mg/dl) (n = 182/207)	1.24 (0.89–2.84)
Leukocyte count at switch (*10^9^/l) (n = 187/207)	6.6 (5.5–8.3)
Co-trimoxaxole use at switch (n = 194/207)	
• None	41 (21%)
• Prophylactic dose (480 mg/day)	130 (67%)
• Therapeutic dose (1920 mg/day)	23 (12%)
Cyc start dose (mg/kg/day) (n = 191/207)	1.7 (0.4)
Duration of cyc therapy (months) (n = 206/207)	5 (4–6)
Prednisolone switch dose (mg/day)(n = 188/207)	12.5 (8.1–20.0)
Azathioprine switch dose (mg/kg/day) (n = 195/207)	1.4 (0.5)
Duration of azathioprine therapy (months) (n = 204/207)	17 (7–24)
Follow-up time (months)	54 (32–60)
*TPMT* genotype	
• No variant (*1/*1)	188 (91%)
• TPMT *1/*3A	16 (8%)
• TPMT *1/*3C	3 (1%)
TPMT activity (nmol/gHb/hr)	80.0 (17.9)

GPA granulomatosis with polyangiitis; MPA microscopic polyangiitis; RLV renal limited vasculitis; PR3 proteinase 3; MPO myeloperoxidase; BVAS Birmingham Vasculitic Activity Score; Cyc cyclophosphamide; TPMT thiopurine methyltransferase

**Fig 2:**
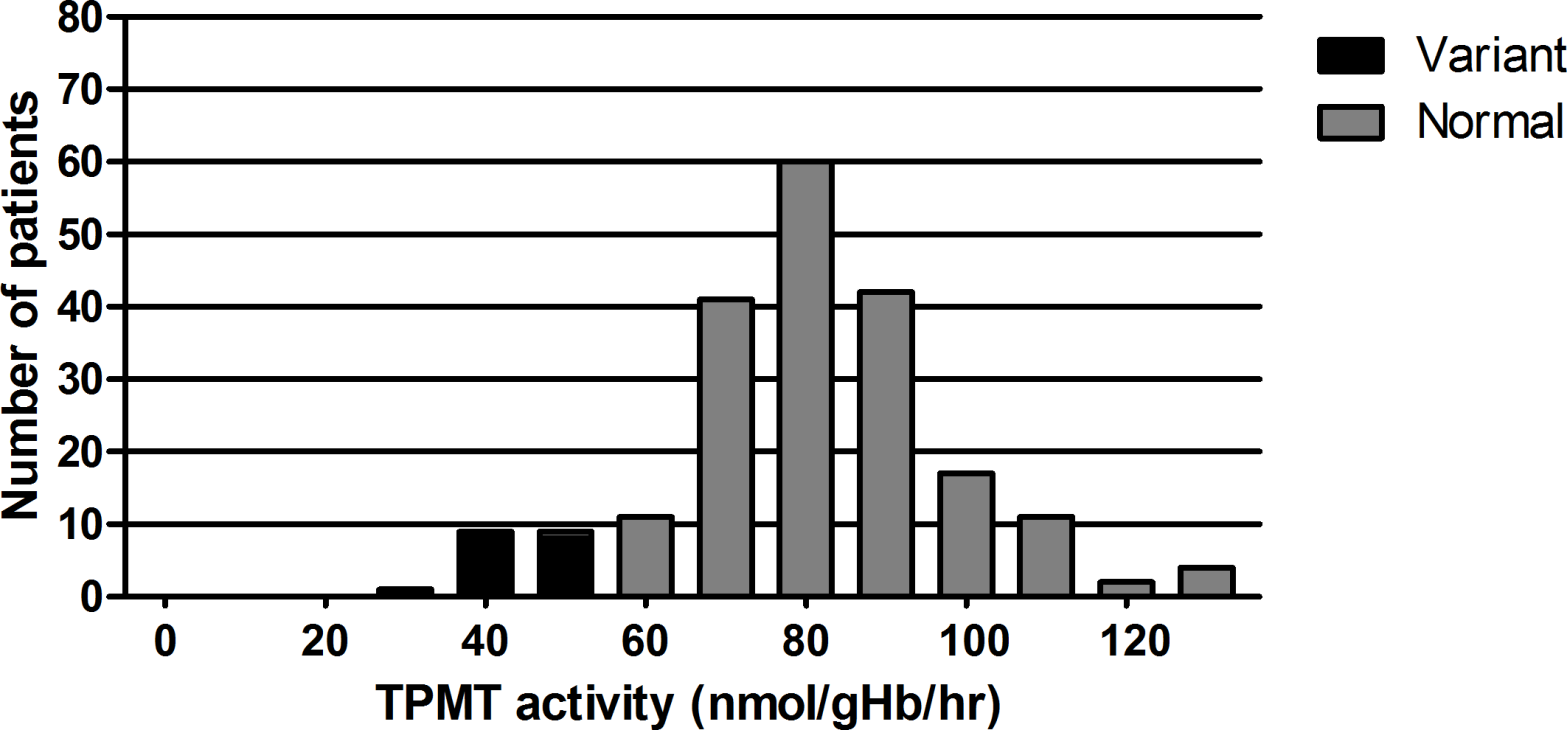
Distribution of TPMT activity. TPMT activity in nmol/gHb/hr for patients with normal (gray) and heterozygous (black) TPMT genotype.

TPMT activity approximated a Gaussian distribution (Fig 2). TPMT activity was significantly lower in carriers of TPMT*3A (43.9; IQR 40.6–49.5 nmol/gHb/hr) and TPMT*3C (43.5 nmol/gHb/hr) compared to patients with a homozygous normal genotype (81.4; IQR 73.5–92.2) nmol/gHb/hr)(P<0.001). TPMT activity was divided in tertiles based on the number of patients. The lowest tertile (T1) contains patients with TPMT activity ≤74.5, the second tertile (T2) contains patients with TPMT activity 74.6–86.4 and the highest tertile (T3) contains patients with TPMT activity ≥86.5 nmol/gHb/hr. None of the characteristics differed between *TPMT* genotypes or tertiles of TPMT activity, except azathioprine dose at switch, which was significantly lower in patients with heterozygous *TPMT* genotype (1.0, IQR 0.7–1.4 mg/kg/day) compared to patients with normal genotype (1.5, IQR 1.1–1.8 mg/kg/day) (P = 0.001). Azathioprine starting dose was not significantly related to measurement of TPMT status prior to (n = 68) or after (n = 139) start of azathioprine therapy (P = 0.92), even when specifically analyzing patients with a heterozygous *TPMT* genotype (P = 0.28). As expected from the treatment protocol, azathioprine starting dose showed a strong positive correlation with cyclophosphamide dose at switch (Rho = 0.70, P<0.001). No patients were treated with 6-mercaptopurin.

### Relapse free survival

**Fig 3:**
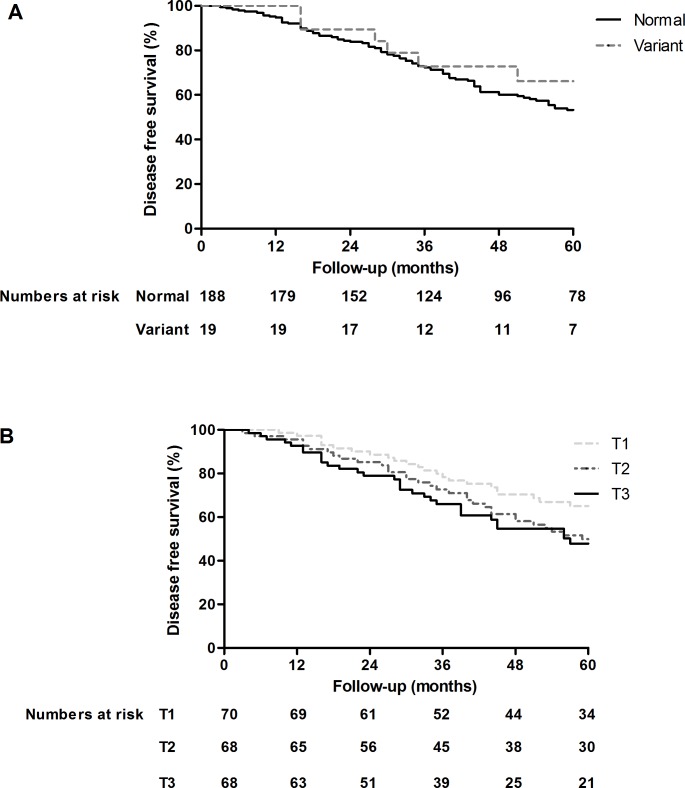
Relapse free survival for *TPMT* genotypes and tertiles of TPMT activity. Relapse free survival after start of cyclophosphamide induction therapy. Upper graph (3A): Variant = heterozygous *TPMT* variant carrier, Normal = normal genotype (wildtype *TPMT*). Lower graph (3B): T1 = lowest tertile, T2 = middle tertile, T3 = highest tertile of TPMT activity.

Within 5 years after diagnosis, 6 of 19 patients (32%) with heterozygous *TPMT* genotype experienced a relapse, compared to 84 of 188 patients (45%) with normal *TPMT* genotype. There was no significant difference in relapse-free survival (P = 0.30) between *TPMT* genotypes (Fig 3A).

Tertiles of TPMT activity showed a negative trend with relapse-free survival (P = 0.07) (Fig 3B). In the lowest tertile (T1), 34% of patients experienced relapse within 5 years, compared to 47% in the middle tertile (T2) and 50% in the highest tertile (T3). Relapse free survival was still not significantly related to *TPMT* genotype (P = 0.39) and tertiles of TPMT activity (P = 0.21) after exclusion of patients intolerant to azathioprine.

In Cox regression, *TPMT* genotypes (P = 0.39), tertiles of TPMT activity (P = 0.24), co-trimoxazole use (P = 0.15), age (P = 0.69) and diagnosis (P = 0.94) were not significantly related to the occurrence of relapse. ANCA specificity (P = 0.003), duration of azathioprine therapy (P<0.001), serum creatinine at baseline (P = 0.007) and leukocyte count at switch (P = 0.001) were significantly associated with relapse. Risk of relapse was higher for PR3-ANCA positive patients (Hazard Ratio (HR)3.1; 95% CI 1.5–6.6), and for patients with a higher leukocyte count after cyclophosphamide induction (HR 1.17; 95% CI 1.07–1.29). Risk of relapse was lower for patients with a longer duration of azathioprine maintenance (HR 0.91; 95%CI 0.87–0.96), and patients with a baseline creatinine >1.24 mg/dl (HR 0.5, 95%CI 0.3–0.8). The interaction [azathioprine duration]*[time] was significant (P = 0.008) and indicated a declining protective effect of azathioprine duration over time (HR 1.002, 95%CI 1.000–1.003). The same variables remained significant after exclusion of patients intolerant to azathioprine (S2 Table).

### Adverse events

In total, 35 patients (16%) were intolerant to azathioprine. 17 patients (8%) had gastro-intestinal complaints, 17 (8%) had a febrile hypersensitivity reaction, and 1 patient (1%) had a rash. Intolerance to azathioprine was not related to *TPMT* genotype (P = 0.11) or tertiles of TPMT activity (P = 0.39). There was no significant difference between *TPMT* genotypes in occurrence of mild or moderate leukopenia (Table 2). Occurrence of mild or moderate leukopenia also did not significantly differ between tertiles of TPMT activity (Table 3).

The lowest measured leukocyte count was 1.4*10^^9^/l. Two patients had concomitant infections (PCP pneumonia and CMV antigenemia, candida stomatitis and PCP pneumonia, respectively). In 12 patients with moderate leukopenia, azathioprine dose was reduced and in 9 patients azathioprine was (temporarily) discontinued. In all except 3 patients, moderate leukopenia was incidental. In the others durations were 5, 7 and 35 days before leukocyte counts were >3.0*10^9^/l.

**Table 2:** Adverse events in relation to *TPMT* genotype.

Adverse events	All n(%)	Variant n(%)	Normal n(%)	P*
All azathioprine tolerant patients	172	15	157	
Missing data on leukopenia	8	0	8	0.79
Leukopenia	75 (46)	6 (40)	69 (46)	
• Mild leukopenia (<4*10^9^/l)	54 (33)	4 (27)	50 (34)	>0.99
• Moderate leukopenia (<3*10^9^/l)	21 (13)	2 (13)	19 (13)	
Missing data on macrocytic anemia	10	0	10	0.28
Macrocytic anemia	74 (46)	9 (60)	65 (44)	
Missing data on hepatotoxicity	8	0	8	0.26
Hepatotoxicity	26 (16)	4 (27)	22 (15)	
Missing data on infections	11	1	10	0.40
Infection	62 (39)	7 (50)	55 (37)	

Number of patients experiencing adverse events. All analyses (except for intolerance) have been done only in patients who were not intolerant to azathioprine (n = 172). All = all patients. Variant = heterozygous *TPMT* variant carrier, Normal = normal *TPMT* genotype.

*Compared between *TPMT* genotypes.

**Table 3:** Adverse events in relation to TPMT activity.

Adverse events	All n(%)	T1 n (%)	T2 n (%)	T3 n (%)	P*
All azathioprine tolerant patients	172	57	55	60	
Missing data on leukopenia	8	1	1	6	0.82
Leukopenia	75 (46)	27(48)	23 (43)	25 (46)	
• Mild leukopenia (<4*10^9^/l)	54 (33)	17 (31)	20 (37)	17 (31)	0.13
• Moderate leukopenia (<3*10^9^/l)	21 (13)	10 (18)	3 (6)	8 (15)	
Missing data on macrocytic anemia	10	2	2	6	0.11
Macrocytic anemia	74 (46)	29 (53)	18 (34)	27 (50)	
Missing data on hepatotoxicity	8	1	1	6	0.48
Hepatotoxicity	26 (16)	11 (20)	6 (11)	9 (17)	
Missing data on infections	11	3	1	7	0.90
Infection	62 (39)	22 (41)	21 (39)	19 (36)	

Number of patients experiencing adverse events. All analyses (except for intolerance) have been done only in patients who were not intolerant to azathioprine (n = 172). All = all patients. T1 = first tertile, T2 = second tertile, T3 = third tertile of TPMT activity.

*Compared between tertiles of TPMT activity.

Azathioprine starting dose was not significantly different between patients with (median 1.6; IQR 1.2–1.8 mg/kg) and without (median 1.4; IQR 0.9–1.8 mg/kg) mild leukopenia (P = 0.08), neither between patients with (median 1.5; IQR 1.1–1.7 mg/kg) or without (median 1.5; IQR 1.1–1.8) moderate leukopenia (P = 0.65). The same goes for patients with and without macrocytic anemia (P = 0.09), liver toxicity (P = 0.26) and infections (P = 0.69).

In logistic regression, TPMT genotype and activity were not significantly related to leukopenia during azathioprine therapy. Prednisolone dose and cyclophosphamide dose at switch were also not significant. Leukocyte count at switch (*i*.*e*. at the end of induction therapy with cyclophosphamide) remained in the model as a significant predictor of leukopenia (P<0.001), as well as azathioprine dose at switch (P = 0.04) with a higher risk of leukopenia during azathioprine therapy in patients with a lower leukocyte count at the end of cyclophosphamide therapy (Odds Ratio (OR) 0.54; 95% CI 0.43–0.68), and for patients with a higher starting dose of azathioprine (OR 2.2; 95% CI 1.0–4.6). See also S3 Table.

**Fig 4:**
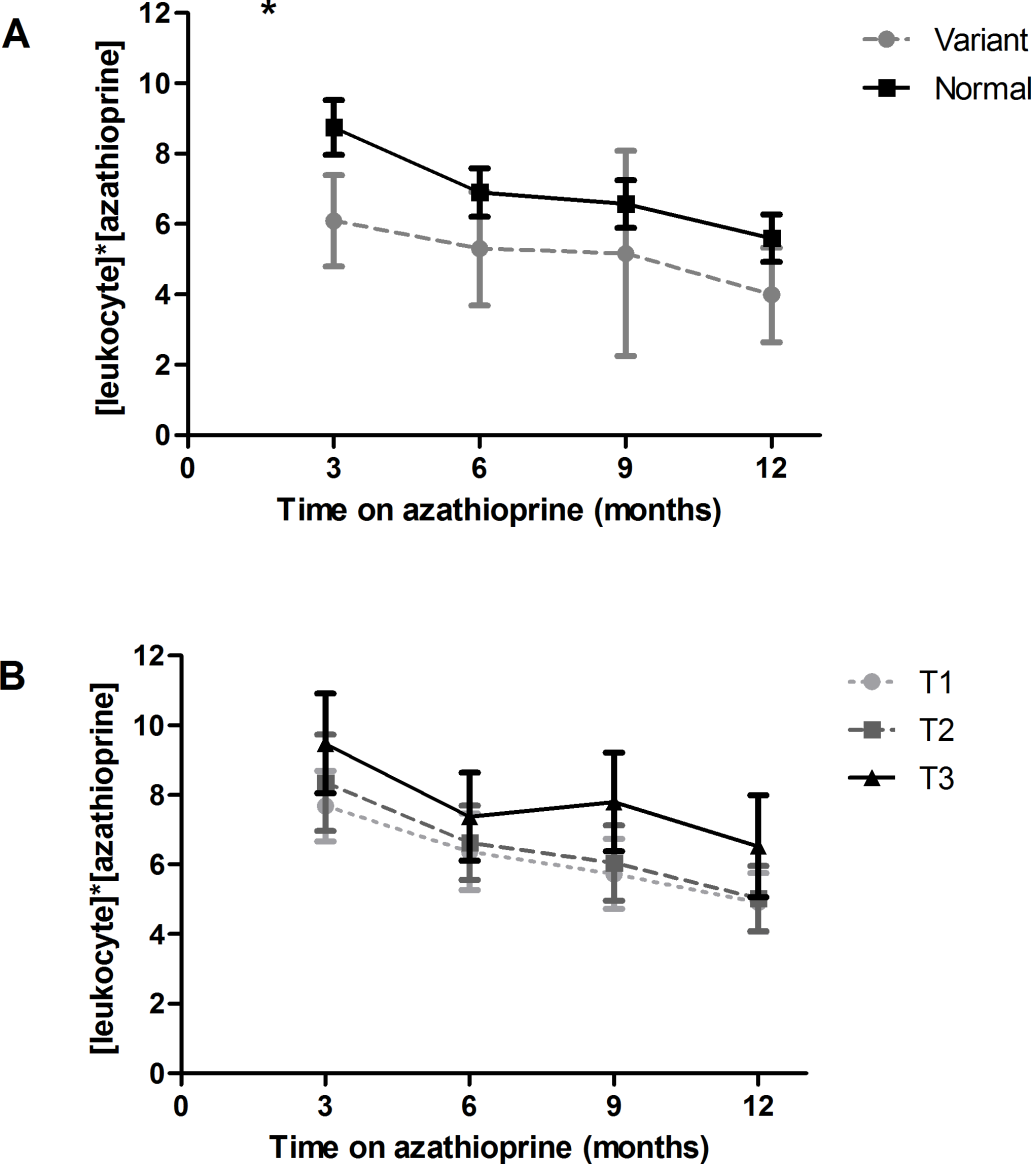
[leuko]*[aza] product over time for *TPMT* genotypes and tertiles of TPMT activity. [leukocyte]*[azathioprine] product 3,6,9 and 12 months after switch to azathioprine. *P<0.05. Upper graph (4A): Variant = heterozygous *TPMT* variant carrier; Normal = normal genotype (wildtype *TPMT*). Lower graph (4B): T1 = lowest tertile, T2 = middle tertile, T3 = highest tertile of TPMT activity.

Leukocyte counts 3, 6, 9 and 12 months after switch to azathioprine were not significantly different between *TPMT* genotypes or tertiles of TPMT activity. The [leukocyte]*[azathioprine] product was significantly lower for heterozygous patients 3 months after switch (P = 0.03) but not at later time points (Fig 4A). The [leukocyte]*[azathioprine] product did not significantly differ between tertiles of TPMT activity (Fig 4B).

In multivariate linear regression, after correction for prednisolone dose, *TPMT* genotype was still not a significant predictor of leukocyte count or [leukocyte]*[azathioprine] product at any time point. Tertiles of TPMT activity, on the other hand, were positively related to leukocyte counts 3 months (P<0.001; b (regression coefficient of predictor) = 0.74; 95% CI 0.35–1.14) and 9 months after switch (P = 0.04; b = 0.36; 95% CI 0.02–0.70) after correction for prednisolone dose. Multivariate linear regression also showed a significant association of TPMT activity tertiles with the [leukocyte]*[azathioprine] product 3 (P = 0.003; b = 1.20; 95% CI 0.41–2.00), 9 (P = 0.01; b = 0.94; 95% CI 0.20–1.69) and 12 months after switch (P = 0.02; b = 0.76; 95% CI 0.12–1.41). This indicates higher [leukocyte]*[azathioprine] product and therefore lower sensitivity for azathioprine-induced leukopenia in patients with higher TPMT activity. Prednisolone dose showed a significant positive association with leukocyte counts and the [leukocyte]*[azathioprine] product at every time point (P≤0.001).

*TPMT* genotype was not related to occurrence of macrocytic anemia, liver toxicity, infection, or intolerance to azathioprine (Table 2). Occurrence of these adverse events was also not significantly different between tertiles of TPMT activity (Table 3).

## Discussion

In this study, we found no significant association of *TPMT* genotype and TPMT activity with relapse free survival. TPMT genotype and activity were not related to occurrence of azathioprine related adverse events. Leukocyte counts at the end of cyclophosphamide induction therapy were significantly associated with both relapse free survival and occurrence of leukopenia during azathioprine maintenance therapy.

Because azathioprine therapy is associated with a risk of potentially severe adverse events such as bone marrow toxicity, several studies have focused on TPMT genotypes and activity as predictors of these adverse events.[8,15] It has been established mainly in IBD patient populations that patients with a heterozygous *TPMT* genotype and lower TPMT activity are at increased risk of developing adverse events,[8] and that pretesting for TPMT has a beneficial effect specifically in the group of patients with one or several *TPMT* variants.[14] Although we found that a lower TPMT activity was associated with a lower [leukocyte]*[azathioprine] product, indicating an increased sensitivity to azathioprine-induced leukopenia, we did not find an association of *TPMT* genotype and activity with bone marrow toxicity in our population of AAV patients. This might be explained by the fact that AAV patients do not receive azathioprine as the main treatment drug, but as maintenance therapy after induction therapy with cyclophosphamide.[4] The effect of cyclophosphamide on bone marrow toxicity during azathioprine therapy may be greater than the effect of TPMT, as evidenced by the strong association of leukocyte counts after cyclophosphamide treatment with both relapse free survival and leukopenia during azathioprine therapy in this study, and the lack of a significant difference in azathioprine starting dose between patients with and without leukopenia. Another explanation may be that leukocyte counts after cyclophosphamide therapy reflect an overall bone marrow susceptibility to the effects of both drugs.

The frequency of leukopenia in or population was relatively high compared to previous reports, such as in the CYCAZAREM trial (30%).[5] The first reason for this is that patients with leukopenia at the start of azathioprine therapy were also scored as having leukopenia during azathioprine therapy. The second reason is that any leukopenia during the full duration of azathioprine therapy was scored, compared to only the first 15 months after switch in the CYCAZAREM trial.[5] When counting only patients that developed leukopenia within 15 months after start of azathioprine, the frequency of leukopenia (<4.0*10^9^/l) in our population was 31%, similar to the frequency previously reported.[5]

Theoretically, patients with lower TPMT activity can achieve a higher efficacy of azathioprine.[8,23] Some studies indeed found an association of TPMT activity with clinical response.[24,25] In a recently published RCT, adjusting azathioprine dose based on *TPMT* genotype did not result in a difference in treatment response between intervention and control groups.[14] Although we found that patients with *TPMT* variant alleles and patients with lower TPMT activity had a higher relapse free survival, these differences were not significant, especially when taking other predictors of relapse into account. Interestingly, higher leukocyte counts after cyclophosphamide therapy were a strong predictor of relapse. This indicates that response to cyclophosphamide may be a stronger predictor of clinical efficacy than TPMT.

This study has several limitations. First, although 207 patients is an impressive number for a single center study on a rare disease such as AAV, the sample size may be insufficient to detect relevant associations with sufficient power. This is especially true for the analyses on *TPMT* genotype, since there are only 19 patients with a heterozygous *TPMT* genotype in our study. Second, treating physicians were not blinded to a patient’s TPMT genotype and activity. On the other hand, adjustment of azathioprine dose based on TPMT status was not included in the treatment protocol, and the initial azathioprine dose of patients with heterozygous *TPMT* genotype did not differ between patients whether their TPMT genotype and activity were measured before or after azathioprine therapy. Third, the study was conducted in a tertiary referral center, with some patients receiving part of their follow-up elsewhere. This resulted in missing values on adverse effects of 11 patients. The baseline characteristics and induction treatment of these patients did not significantly differ from those of patients with follow-up during azathioprine therapy. Lastly, the ethnicity for patients was not registered, although over 95% of patients in our study population are estimated to be Caucasian. As we only genotyped for TPMT variants common in Caucasians, some non-Caucasian patients might have reduced TPMT activity resulting from an untyped *TPMT* variant. This could theoretically result in underestimation of the effects from *TPMT* genotype.

The study also has several strengths. It was performed in a single center and all patients were treated according to the same protocol, thereby eliminating between-center differences in treatment and followup measurements. Also, compared to an earlier study on TPMT genotype and activity in AAV patients from our population,[18] this study has a larger sample size (207 compared to 108), has a longer duration of follow-up and includes multivariable analyses to account for induction treatment and other factors influencing disease free survival and risk of adverse events.

In conclusion, TPMT genotype and activity were not related to azathioprine efficacy and toxicity in our retrospective cohort of AAV patients receiving azathioprine maintenance therapy. Response to cyclophosphamide, on the other hand, may have a stronger predictive value on these outcomes. This should be confirmed in a sufficiently large multicenter study.

## Supporting information

S1 Table Tapering scheme for prednisolone. † Start tapering earlier when in full remission for 2weeks, <6wks of therapy.
(DOCX)

S2 Table Cox regression for 5 year relapse free survival. Cox regression analysis for 5 year/60 month relapse free survival for non-azathioprine intolerant patients (n = 172). Variables for the final model were selected using a forward stepwise method (inclusion if univariate P<0.05, exclusion if multivariate P>0.1). ANCA specificity, duration of azathioprine therapy, creatinine at baseline and leukocyte count after cyclophosphamide induction therapy were significantly associated with risk of relapse. *P<0.05; **P<0.01; ***P<0.001.
(DOCX)

S3 Table Logistic regression for risk of leukopenia. Logistic regression for risk of leukopenia (leukocyte count <4.0*109/l) for non-intolerant patients (n = 172). Variables for the final model were selected using a forward stepwise method (inclusion if univariate P<0.05, exclusion if multivariate P>0.1). A higher leukocyte count after cyclophosphamide induction therapy was associated with a lower risk of leukopenia, and a higher starting dose of azathioprine was associated with a higher risk of leukopenia during azathioprine therapy. *P<0.05; **P<0.01; ***P<0.001.
(DOCX)

## References

[1] Jennette JC. Overview of the 2012 revised International Chapel Hill Consensus Conference nomenclature of vasculitides. Clin Exp Nephrol 2013 10;17(5):603–606. doi: 10.1007/s10157-013-0869-6 24072416 10.1007/s10157-013-0869-6 PMC4029362

[2] Stone JH, Merkel PA, Spiera R, Seo P, Langford CA, Hoffman GS, et al Rituximab versus cyclophosphamide for ANCA-associated vasculitis. N Engl J Med 2010 7 15;363(3):221–232. doi: 10.1056/NEJMoa0909905 20647199 10.1056/NEJMoa0909905 PMC3137658

[3] Wall N, Harper L. Complications of long-term therapy for ANCA-associated systemic vasculitis. Nat Rev Nephrol 2012 9;8(9):523–532. doi: 10.1038/nrneph.2012.107 22664736 10.1038/nrneph.2012.107

[4] Kallenberg CG. Pathogenesis and treatment of ANCA-associated vasculitides. Clin Exp Rheumatol 2015 Sep-Oct;33(4 Suppl 92):11–14. 26457917

[5] Jayne D, Rasmussen N, Andrassy K, Bacon P, Tervaert JW, Dadoniene J, et al A randomized trial of maintenance therapy for vasculitis associated with antineutrophil cytoplasmic autoantibodies. N Engl J Med 2003 7 3;349(1):36–44. doi: 10.1056/NEJMoa020286 12840090 10.1056/NEJMoa020286

[6] Pagnoux C, Mahr A, Hamidou MA, Boffa JJ, Ruivard M, Ducroix JP, et al Azathioprine or methotrexate maintenance for ANCA-associated vasculitis. N Engl J Med 2008 12 25;359(26):2790–2803. doi: 10.1056/NEJMoa0802311 19109574 10.1056/NEJMoa0802311

[7] Roberts RL, Barclay ML. Current relevance of pharmacogenetics in immunomodulation treatment for Crohn's disease. J Gastroenterol Hepatol 2012 10;27(10):1546–1554. doi: 10.1111/j.1440-1746.2012.07220.x 22741564 10.1111/j.1440-1746.2012.07220.x

[8] Moon W, Loftus EV Jr. Review article: recent advances in pharmacogenetics and pharmacokinetics for safe and effective thiopurine therapy in inflammatory bowel disease. Aliment Pharmacol Ther 2016 2 14. 10.1111/apt.13559 26876431

[9] Yates CR, Krynetski EY, Loennechen T, Fessing MY, Tai HL, Pui CH, et al Molecular diagnosis of thiopurine S-methyltransferase deficiency: genetic basis for azathioprine and mercaptopurine intolerance. Ann Intern Med 1997 4 15;126(8):608–614. 9103127 10.7326/0003-4819-126-8-199704150-00003

[10] Weinshilboum RM, Sladek SL. Mercaptopurine pharmacogenetics: monogenic inheritance of erythrocyte thiopurine methyltransferase activity. Am J Hum Genet 1980 9;32(5):651–662. 7191632 PMC1686086

[11] Gisbert JP, Gomollon F. Thiopurine-induced myelotoxicity in patients with inflammatory bowel disease: a review. Am J Gastroenterol 2008 7;103(7):1783–1800. doi: 10.1111/j.1572-0241.2008.01848.x 18557712 10.1111/j.1572-0241.2008.01848.x

[12] Higgs JE, Payne K, Roberts C, Newman WG. Are patients with intermediate TPMT activity at increased risk of myelosuppression when taking thiopurine medications? Pharmacogenomics 2010 2;11(2):177–188. doi: 10.2217/pgs.09.155 20136357 10.2217/pgs.09.155

[13] Dong XW, Zheng Q, Zhu MM, Tong JL, Ran ZH. Thiopurine S-methyltransferase polymorphisms and thiopurine toxicity in treatment of inflammatory bowel disease. World J Gastroenterol 2010 7 7;16(25):3187–3195. doi: 10.3748/wjg.v16.i25.3187 20593505 10.3748/wjg.v16.i25.3187 PMC2896757

[14] Coenen MJ, de Jong DJ, van Marrewijk CJ, Derijks LJ, Vermeulen SH, Wong DR, et al Identification of Patients With Variants in TPMT and Dose Reduction Reduces Hematologic Events During Thiopurine Treatment of Inflammatory Bowel Disease. Gastroenterology 2015 10;149(4):907–17.e7. doi: 10.1053/j.gastro.2015.06.002 26072396 10.1053/j.gastro.2015.06.002

[15] Liu YP, Xu HQ, Li M, Yang X, Yu S, Fu WL, et al Association between Thiopurine S-Methyltransferase Polymorphisms and Azathioprine-Induced Adverse Drug Reactions in Patients with Autoimmune Diseases: A Meta-Analysis. PLoS One 2015 12 3;10(12):e0144234 doi: 10.1371/journal.pone.0144234 26633017 10.1371/journal.pone.0144234 PMC4669175

[16] Newman WG, Payne K, Tricker K, Roberts SA, Fargher E, Pushpakom S, et al A pragmatic randomized controlled trial of thiopurine methyltransferase genotyping prior to azathioprine treatment: the TARGET study. Pharmacogenomics 2011 6;12(6):815–826. doi: 10.2217/pgs.11.32 21692613 10.2217/pgs.11.32

[17] Gisbert JP, Luna M, Mate J, Gonzalez-Guijarro L, Cara C, Pajares JM. Choice of azathioprine or 6-mercaptopurine dose based on thiopurine methyltransferase (TPMT) activity to avoid myelosuppression. A prospective study. Hepatogastroenterology 2006 May-Jun;53(69):399–404. 16795981

[18] Stassen PM, Derks RP, Kallenberg CG, Stegeman CA. Thiopurinemethyltransferase (TPMT) genotype and TPMT activity in patients with anti-neutrophil cytoplasmic antibody-associated vasculitis: relation to azathioprine maintenance treatment and adverse effects. Ann Rheum Dis 2009 5;68(5):758–759. doi: 10.1136/ard.2008.097667 19366895 10.1136/ard.2008.097667

[19] Luqmani RA, Bacon PA, Moots RJ, Janssen BA, Pall A, Emery P, et al Birmingham Vasculitis Activity Score (BVAS) in systemic necrotizing vasculitis. QJM 1994 11;87(11):671–678. 7820541

[20] Little MA, Nightingale P, Verburgh CA, Hauser T, De Groot K, Savage C, et al Early mortality in systemic vasculitis: relative contribution of adverse events and active vasculitis. Ann Rheum Dis 2010 6;69(6):1036–1043. doi: 10.1136/ard.2009.109389 19574233 10.1136/ard.2009.109389

[21] Kroplin T, Weyer N, Gutsche S, Iven H. Thiopurine S-methyltransferase activity in human erythrocytes: a new HPLC method using 6-thioguanine as substrate. Eur J Clin Pharmacol 1998 5;54(3):265–271. 9681671 10.1007/s002280050457

[22] Bellera CA, MacGrogan G, Debled M, de Lara CT, Brouste V, Mathoulin-Pelissier S. Variables with time-varying effects and the Cox model: some statistical concepts illustrated with a prognostic factor study in breast cancer. BMC Med Res Methodol 2010 3 16;10:20-2288-10-20. 10.1186/1471-2288-10-20 PMC2846954 20233435

[23] Coulthard SA, Hogarth LA, Little M, Matheson EC, Redfern CP, Minto L, et al The effect of thiopurine methyltransferase expression on sensitivity to thiopurine drugs. Mol Pharmacol 2002 7;62(1):102–109. 12065760 10.1124/mol.62.1.102

[24] Ansari A, Hassan C, Duley J, Marinaki A, Shobowale-Bakre EM, Seed P, et al Thiopurine methyltransferase activity and the use of azathioprine in inflammatory bowel disease. Aliment Pharmacol Ther 2002 10;16(10):1743–1750. 12269967 10.1046/j.1365-2036.2002.01353.x

[25] Cuffari C, Dassopoulos T, Turnbough L, Thompson RE, Bayless TM. Thiopurine methyltransferase activity influences clinical response to azathioprine in inflammatory bowel disease. Clin Gastroenterol Hepatol 2004 5;2(5):410–417. 15118980 10.1016/s1542-3565(04)00127-2

