# Supplementary material for: Thiopurine methyltransferase genotype and activity cannot predict outcomes of azathioprine maintenance therapy for antineutrophil cytoplasmic antibody associated vasculitis: A retrospective cohort study
Source: PLoS One. 2018 Apr 9;13(4):e0195524. doi: 10.1371/journal.pone.0195524 (PMC5890988; doi:10.1371/journal.pone.0195524)
Supplement: S1 Table — † Start tapering earlier when in full remission for 2weeks, <6wks of therapy. (DOCX) [file pone.0195524.s001.docx]

**S1 table. Tapering scheme for prednisolone**

| **Time from start of therapy (weeks)** | **Prednisolone daily dose (mg)** |
| --- | --- |
| 0 – 6† | 60 |
| 6 – 12 | Reduce 10 mg per 2 weeks up to 30 mg |
| 12 – 18 | Reduce 5 mg per 2 weeks up to 15 mg |
| 18 – 28 | Reduce 2,5 mg per 2 weeks up to 0 |
| > 28 | Stop |

- **Start tapering earlier when in full remission for 2weeks, <6wks of therapy**
