# Supplementary material for: Thiopurine methyltransferase genotype and activity cannot predict outcomes of azathioprine maintenance therapy for antineutrophil cytoplasmic antibody associated vasculitis: A retrospective cohort study
Source: PLoS One. 2018 Apr 9;13(4):e0195524. doi: 10.1371/journal.pone.0195524 (PMC5890988; doi:10.1371/journal.pone.0195524)
Supplement: S2 Table — Cox regression analysis for 5 year/60 month relapse free survival for non-azathioprine intolerant patients (n = 172). Variables for the final model were selected using a forward stepwise method (inclusion if univariate P<0.05, exclusion if multivariate P>0.1). ANCA specificity, duration of azathioprine therapy, creatinine at baseline and leukocyte count after cyclophosphamide induction therapy were significantly associated with risk of relapse. *P<0.05; **P<0.01; ***P<0.001. (DOCX) [file pone.0195524.s002.docx]

**S2 Table. Cox regression for 5 year relapse free survival**

| **Variable** | **P-value** | **HR + 95% CI** |
| --- | --- | --- |
| **Included in final model** |  |  |
| Creatinine level at baseline (<=110 or >110 umol/l) | 0.01 (*) | 0.5 (0.3-0.9) |
| Leukocyte count at switch | 0.001 (**) | 1,18 (1,07-1,31) |
| ANCA (PR3 vs MPO/other/negative) | 0.007 (**) | 3.1 (1.4-6.9) |
| Duration of azathioprine therapy (months) | <0.001 (***) | 0.86 (0.79-0.93) |
| Time * duration of azathioprine therapy | 0.002 (**) | 1.003 (1.001-1.005) |
| **Not included in final model** |  |  |
| TPMT genotype | 0.66 | - |
| Tertiles of TPMT activity | 0.41 | - |
| Age | 0.91 | - |
| Diagnosis (GPA/MPA/NCGN) | 0.69 | - |
| Co-trimoxazole dose at switch (high/low/none) | 0.38 | - |

**Cox regression analysis for 5 year/60 month relapse free survival for non-azathioprine intolerant patients (n=172). Variables for the final model were selected using a forward stepwise method (inclusion if univariate P<0.05, exclusion if multivariate P>0.1). ANCA specificity, duration of azathioprine therapy, creatinine at baseline and leukocyte count after cyclophosphamide induction therapy were significantly associated with risk of relapse. *P<0.05; **P<0.01; ***P<0.001.**
