# Supplementary material for: Thiopurine methyltransferase genotype and activity cannot predict outcomes of azathioprine maintenance therapy for antineutrophil cytoplasmic antibody associated vasculitis: A retrospective cohort study
Source: PLoS One. 2018 Apr 9;13(4):e0195524. doi: 10.1371/journal.pone.0195524 (PMC5890988; doi:10.1371/journal.pone.0195524)
Supplement: S3 Table — Logistic regression for risk of leukopenia (leukocyte count <4.0*109/l) for non-intolerant patients (n = 172). Variables for the final model were selected using a forward stepwise method (inclusion if univariate P<0.05, exclusion if multivariate P>0.1). A higher leukocyte count after cyclophosphamide induction therapy was associated with a lower risk of leukopenia, and a higher starting dose of azathioprine was associated with a higher risk of leukopenia during azathioprine therapy. *P<0.05; **P<0.01; ***P<0.001. (DOCX) [file pone.0195524.s003.docx]

**S3 Table. Logistic regression for risk of leukopenia**

| **Variable** | **P-value** | **OR + 95% CI** |
| --- | --- | --- |
| **Included in final model** |  |  |
| Azathioprine switch dose | 0.04(*) | 2.2 (1.0-4.6) |
| Leukocyte count at switch | <0.001 (***) | 0.54 (0.43-0.68) |
| **Not included in final model** |  |  |
| TPMT genotype | 0.85 | - |
| Tertiles of TPMT activity | 0.83 | - |
| Cyclophosphamide switch dose | 0.44 | - |
| Prednisolone switch dose | 0.14 | - |

**Logistic regression for risk of leukopenia (leukocyte count <4.0*109/l) for non-intolerant patients (n=172). Variables for the final model were selected using a forward stepwise method (inclusion if univariate P<0.05, exclusion if multivariate P>0.1). A higher leukocyte count after cyclophosphamide induction therapy was associated with a lower risk of leukopenia, and a higher starting dose of azathioprine was associated with a higher risk of leukopenia during azathioprine therapy. *P<0.05; **P<0.01; ***P<0.001.**
